# Off-label use of omalizumab in a 6-year-old child with severe atopic dermatitis

**DOI:** 10.5339/qmj.2022.fqac.21

**Published:** 2022-03-28

**Authors:** Raed Alzyoud

**Affiliations:** Pediatric Immunology, Allergy, and Rheumatology Division, Queen Rania Children's Hospital, Amman-Jordan. E-mail: raedalzyoud@gmail.com

**Keywords:** off-label use, omalizumab, refractory AD

## Abstract

Background: Atopic dermatitis (AD) is a common chronic inflammatory skin disorder that affects up to 3% of adults and 15%-20% of children across the world. Although the mainstay of treatment is topical corticosteroids and/or calcineurin inhibitors, a majority of patients will not achieve a control of their condition and will need systemic treatment with steroids and immunosuppressants (1). Omalizumab is a humanized anti-IgE antibody licensed by the European Medical Agency for treatment of severe allergic asthma and spontaneous chronic urticaria in children older than 6 and 12 years, respectively; and a promising efficacy of omalizumab in the treatment of severe refractory AD has been shown by the ADAPT randomized clinical trial (2).

Case report: A 6-year-old male patient being treated and followed at a pediatric dermatology clinic for a diagnosis of AD since the age of 3 years, showed unresponsive disease to different topical treatment options and non-pharmacological measures. The patient was referred to our clinic for systemic treatment when his SCORAD index was 70/103. At our immunology clinic, we did an extensive workup to unveil a comorbid or underlying disease. His labs showed high IgE levels at 2500 IU/mL, hypereosinophilia, and normal IgA, IgM, and lymphocyte subsets. We started him on different lines of immunosuppressants, including cyclosporine, prednisolone, tacrolimus, and intensive topical treatment; however, the patient did not show any noticeable response for 2 years. We then started him on omalizumab 300 mg twice weekly, and the SCORAD index dropped to 57 at 12 weeks and 55 at 18 weeks. The drug is still well tolerated, and we were able to stop systemic steroids at 15 weeks of treatment.

Conclusion: Off-label use of omalizumab in a 6-year-old child with refractory severe AD was effective and safe in controlling the disease even though conventional systemic immunosuppressants proved ineffective.

